# XBSeq2: a fast and accurate quantification of differential expression and differential polyadenylation

**DOI:** 10.1186/s12859-017-1803-9

**Published:** 2017-10-03

**Authors:** Yuanhang Liu, Ping Wu, Jingqi Zhou, Teresa L. Johnson-Pais, Zhao Lai, Wasim H. Chowdhury, Ronald Rodriguez, Yidong Chen

**Affiliations:** 1 Greehey Children’s Cancer Research Institute, University of Texas Health Science Center at San Antonio, San Antonio, TX USA; 2 Department of Cellular and Structure Biology, University of Texas Health Science Center at San Antonio, San Antonio, TX USA; 3 Department of Urology, University of Texas Health Science Center at San Antonio, San Antonio, TX USA; 4 Cornell university, Ithaca, NY USA; 5 Department of Epidemiology & Biostatistics, University of Texas Health Science Center at San Antonio, San Antonio, TX USA

**Keywords:** Differential expression analysis, XBSeq, XBSeq2, Alternative polyadenylation, RNA-seq

## Abstract

**Background:**

RNA sequencing (RNA-seq) is a high throughput technology that profiles gene expression in a genome-wide manner. RNA-seq has been mainly used for testing differential expression (DE) of transcripts between two conditions and has recently been used for testing differential alternative polyadenylation (APA). In the past, many algorithms have been developed for detecting differentially expressed genes (DEGs) from RNA-seq experiments, including the one we developed, XBSeq, which paid special attention to the context-specific background noise that is ignored in conventional gene expression quantification and DE analysis of RNA-seq data.

**Results:**

We present several major updates in XBSeq2, including alternative statistical testing and parameter estimation method for detecting DEGs, capacity to directly process alignment files and methods for testing differential APA usage. We evaluated the performance of XBSeq2 against several other methods by using simulated datasets in terms of area under the receiver operating characteristic (ROC) curve (AUC), number of false discoveries and statistical power. We also benchmarked different methods concerning execution time and computational memory consumed. Finally, we demonstrated the functionality of XBSeq2 by using a set of in-house generated clear cell renal carcinoma (ccRCC) samples.

**Conclusions:**

We present several major updates to XBSeq. By using simulated datasets, we demonstrated that, overall, XBSeq2 performs equally well as XBSeq in terms of several statistical metrics and both perform better than DESeq2 and edgeR. In addition, XBSeq2 is faster in speed and consumes much less computational memory compared to XBSeq, allowing users to evaluate differential expression and APA events in parallel. XBSeq2 is available from Bioconductor: http://bioconductor.org/packages/XBSeq/

**Electronic supplementary material:**

The online version of this article (doi:10.1186/s12859-017-1803-9) contains supplementary material, which is available to authorized users.

## Background

Next generation sequencing (NGS) technologies have revolutionized biomedical research. RNA sequencing, different from microarray technology, offers high resolution and has been widely used for transcriptome studies, such as, alternative splicing forms detection, allele-specific expression profiling, alternative polyadenylation site identification and most commonly, differential expression (DE) of transcripts between two conditions (e.g. tumor vs normal).

The abundance level of a transcript is expected to be directly correlated with the number of sequenced fragments that map to that transcript as measured by RNA-seq. Because of this unique characteristic, DE testing methods developed for microarray technology may not be appropriate if directly adopted for RNA-seq. In recent years, various efforts have been made to develop statistical methods for identifying DEGs between two conditions. Poisson and negative binomial models are two most commonly used statistical models among all the statistical methods developed for DE analysis [1–3]. The main differences of different DE algorithms lie in the way they estimate dispersions and particular statistic used for inference. For instance, DESeq2 [4], the latest version of DESeq [2], uses a shrinkage based method for estimation of dispersion which improves stability. Then Wald test or likelihood ratio test is applied to assess significance. edgeR-robust [5], the latest version of edgeR [3], moderates dispersion estimates toward a trended-by-mean estimate. Then likelihood ratio test is also used to assess statistical significance. Recent comparative studies have shown that no single method dominates broad spectrum of scenarios [6, 7]. However, It is worthy of noting that none of the abovementioned methods take into consideration of reads that align to non-exonic regions of the genome as proposed in our earlier study [8].

Alternative polyadenylation (APA) is a widespread mechanism, where alternative poly(A) sites are used by a gene to encode multiple mRNA transcripts of different 3′ untranslated region (UTR) lengths [9]. Approximately 70% of known human genes have been identified with multiple Poly(A) sites in their 3’UTR regions [10], which significantly contributes to transcriptome diversity. APA events affect the fate of mRNA in several ways, for instance, by altering the binding sites of RNA binding proteins and miRNAs. Experimental methods utilizing sequencing technology to quantify relative usage of APA are still under development [11, 12], while it was not known whether RNA-seq, a routine method used for gene expression quantification, could be applied directly to infer APA usage in the past. Recently, several computational methods have been developed for analyzing APA usage using RNA-seq datasets [13, 14], which demonstrates the potential of using RNA-seq for identification of APA events.

Previously we developed an algorithm XBSeq for testing differential expression of RNA-seq, where non-exonic mapped reads are used to model background noise for RNA-seq. To significantly increase the processing speed and functionality, here we provide an updated version: XBSeq2, which include: 1) Updated background annotation file; 2) Functionality to directly process alignment files (.bam files) using featureCounts [15]; 3) Alternative parameter estimation by using Maximum likelihood estimation (MLE); 4) Alternative statistical test for differential expression by using beta distribution approximation; and 5) Incorporation of roar [14] for testing differential APA usage.

## Methods

### Direct processing of bam files using featureCounts

One of the essential step after genome alignment for RNA-seq is the read summarization, or in other words, expression quantification. One of the read summarization algorithm, HTSeq [16], a python package and probably the most widely used program for read summarization, are commonly performed separately in the LINUX environment. To consolidate expression quantification and DE analysis into R environment, we utilize a fast implementation of featureCounts as described below. Similar to featureCounts, summarizeOverlaps, a function from GenomicRanges package [17], also enables user to directly carry out read summarization procedure within R environment.

featureCounts is a read summarization program that can be used for reads generated from RNA or DNA sequencing technologies and it implements highly efficient chromosome hashing and feature blocking techniques that make it considerably faster in speed and consume less computational memory [15]. Previous study has shown that, compared to some other read summarization programs, featureCounts has a similar summarization accuracy but is proven to be much faster and more memory efficient. Currently, featureCounts is available within Subread program [18] and Rsubread package from Bioconductor. In our implementation, we used the default options for feautreCounts, such that, for example, the reads across overlapping genes will not be counted.

### Poisson-negative binomial model

The read count that align to the exonic regions of gene *i* is made up of two components, underneath true signal *S*_*i*_, which is directly related to real expression intensity of gene *i*, and background noise *B*_*i*_, which is largely due to sequencing error or misalignment. Previously, we have developed an algorithm, XBSeq [8], which provides more accurate detection of differential expression for RNA-seq experiments based on Poisson-negative binomial convolution model. A similar statistical model has also been successfully applied to MBDcap-seq [19]. Basically, we assumed that the true signal *S*_*i*_ (what we want to estimate) follows a negative binomial distribution and background noise *B*_*i*_ (sequencing errors or misalignment, etc.) possesses a Poisson distribution. Then the observed signal (what we typically measured) *X*_*i*_ is a convolution of *S*_*i*_ and *B*_*i*_, which is governed by a Delaporte distribution [20].

1 $$ {\displaystyle \begin{array}{l}{X}_i={S}_i+{B}_i\\ {}{S}_i\sim NB\left({r}_i,{p}_i\right)\\ {}{B}_i\sim Poisson\left({\lambda}_i\right)\end{array}} $$

### Estimation of parameters

The assumption is that background noise *B*_*i*_ and true signal *S*_*i*_ are independent. By default, a non-parametric method was used for parameter estimation. Details regarding non-parametric parameter estimation can be found in our previous publication of XBSeq [8].

When sample size is relatively large (> 10, Additional file 1: Table S2), we provide a new way for estimation of parameters by using the maximum likelihood estimation (MLE). The likelihood function is given by:

2 $$ {\displaystyle \begin{array}{l}L\left({\theta}_i\right)=\prod \limits_{j=1}^mp\left({X}_{ij}|{\alpha}_i,{\beta}_i,{\lambda}_i\right)\cdot \prod \limits_{j=1}^mp\left({B}_{ij}|{\lambda}_i\right)\\ {}=\prod \limits_{j=1}^m\sum \limits_{k=0}^{X_{ij}}\frac{\Gamma \left({\alpha}_i+\mathrm{k}\right){\beta}_i^k{\lambda}_i^{X_{ij}-k}{e}^{-{\lambda}_i}}{\Gamma \left({\alpha}_i\right)k!{\left(1+{\beta}_i\right)}^{\left({\alpha}_i+k\right)}\left({X}_{ij}-k\right)!}\\ {}\kern11.5em \cdot \prod \limits_{j=1}^m\frac{\lambda_i^{B_{ij}}{e}^{-{\lambda}_i}}{B_{ij}!}\end{array}} $$

which has no closed form. We applied Broyden–Fletcher–Goldfarb–Shanno (BFGS) algorithm to estimate the parameters by iterative updating. *α*_*i*_ and *β*_*i*_ are parameters for gamma portion of Delaporte distribution which are related to negative binomial parameters by:

3 $$ {r}_i={\alpha}_i $$

4 $$ {p}_i=1/\left({\beta}_i+1\right) $$

### Differential expression testing

After all parameters have been successfully estimated, differential expression testing between two groups (with read count *x* and *y*) will be carried out using a moderated Fisher’s exact test:

5 $$ p=\frac{\sum_{p\left(a,b\right)\le p\left(x,y\right)}p\left(a,b\right)}{\sum_{all}p\left(a,b\right)} $$

where *a* and *b* are constrained by *a + b = x + y*. This step requires heavy computation when *a* and *b* are relatively large.

Here we also provide one updated way for differential expression testing by using beta distribution approximation when the counts are relatively large. For gene *i* with read count *x* and *y* in two groups, we have:

6 $$ z=x+y $$

7 $$ \mu =z/\left({n}_1+{n}_2\right) $$

Where *n*_1_ and *n*_2_ are number of samples in each condition. The two parameters for beta distribution can then be estimated:

8 $$ \alpha ={n}_1\cdot \mu /\left(1+{n}_1/\mu \right) $$

9 $$ \beta ={n}_2\cdot \mu /\left(1+{n}_2/\mu \right) $$

Then center point is defined as:

10 $$ med= qbeta\left(0.5,\alpha, \beta \right) $$

Where *qbeta* is the quantile function of beta distribution. Then *p* value is calculated by:

11 $$ p={2}^{\ast }{k}^{\alpha -1}{\left(1-k\right)}^{\beta -1}/B\left(\alpha, \beta \right) $$

Where *B(α,β)* is the beta function: *B*(*α*, *β*) = *Γ*(*α*)*Γ*(*β*)/*Γ*(*α* + *β*) and *k* = (*x* + 0.5)/*z* if $\frac{x+0.5}{z}< med$ and *k* = (*x* − 0.5)/*z* if $\frac{x-0.5}{z}> med$.

### Prediction of APA sites

APA sites are predicted by using POLYAR program [21], which applies an Expectation Maximization (EM) approach by using 12 different previously mapped poly(A) signal (PAS) hexamer [22]. The predicted APA sites by POLYAR are classified into three classes, PAS-strong, PAS-medium and PAS-weak. Only APA sites in PAS-strong class are selected to construct final APA annotation. APA annotations for human and mouse genome of different versions have been built and are available to download from github: https://github.com/Liuy12/XBSeq_files

### Testing for differential APA usage

Differential APA usage test is carried out using roar package [14]. Basically, the ratio of expression between the short and longer isoform of the transcript, *m/M* ratio, is firstly estimated by:

12 $$ \frac{m}{M}=\frac{l_{post}{r}_{pre}}{l_{pre}{r}_{post}}-1 $$

Where *l*_*pre*_ is the length of the shorter isoform, *l*_*post*_ is the extra length of the longer isoform, *r*_*pre*_ and *r*_*post*_ are the number of reads map to shorter isoform and the portion only to the longer isoform respectively. Then differential APA usage between the two groups will be carried out using Fisher’s exact test. For groups with multiple samples, every combination of comparisons will be examined and significance will be inferred based on a combined *p* value using Fisher’s method.

### Simulation

In order to evaluate the performance of our updated statistical method using beta approximation, we generated a set of simulated datasets where we can control the differential expression status of each gene. In this study, we simulated true signal *S* from a negative binomial distribution and background noise *B* from a Poisson distribution with parameters estimated from a real RNA-seq dataset. We compared XBSeq2 with XBSeq along with DESeq2 [4] and edgeR [3], two most widely used R packages for testing for differential expression for RNA-seq datasets.

We followed a similar simulation procedure described in our previous paper XBSeq [8]. Simply speaking, 5000 genes were randomly selected with replacement after discarding genes with relatively low mapped reads or larger dispersion (top 10%). The true signal *S* was simulated from a negative binomial distribution with parameters estimated from the 5000 selected genes. 10% of the genes were randomly selected to be differentially expressed with 1.5-fold change. We simulated experiments with 3 samples per group. Background noise *B* was generated in three different scenarios, with different level of dispersion, to examine the performance of different methods in normal and noisy conditions. Background noise with different dispersion levels were simulated from a hybrid model:

13 $$ {B}_{inc}\sim {M}^{\ast } Norm\left(\mu, \sigma \right) $$

where *μ* is from a Poisson distribution *μ* ~ *Poisson*(*λ* + *NF*). In our simulation, we set *M* = 100, *σ* = 3. The noise factor *NF* can be chosen from 0, 7, 20, each represents experiments with low background noise, intermediate background noise and high background noise. Simulations were repeated 100 times and statistical metrics were evaluated based on the average performance.

We evaluated XBSeq2 against several other algorithms for their ability to discriminate between differentially expressed and non-differentially expressed genes in terms of the area under the ROC curve, number of false discoveries, and statistical power. The performance of different methods for genes expressed at high and low levels were also examined to see whether the algorithm is affected by expression intensity of the gene.

### RNA-seq dataset for testing

Tumor and adjacent normal tissues from six clear cell renal cell carcinoma (ccRCC) patients. Were obtained from the UTHSCSA Genitourinary Tissue Bank. Total RNA was used for stranded mRNA-Seq library preparation by following the KAPA Stranded RNA-Seq Kit with RiboErase (HMR) sample preparation guide. RNA-Seq libraries were sequenced with 100 bp paired end sequencing run with Illumina HiSeq 2000 platform. After sequencing procedure, alignment was carried out using BWA and differential expression and differential APA usage testing were carried out using XBSeq2.

### Compare with other algorithms

We compared XBSeq2 (1.3.2) with some other methods including XBSeq (1.2.2), DESeq2 (1.8.2), edgeR (3.10.5). All the analysis and evaluation were carried out using R version 3.2.0 and Bioconductor version 1.20.3.

## Results

### Updates of XBSeq algorithm

Previously, we have developed an algorithm, XBSeq, for detecting differentially expressed genes for RNA-seq datasets by taking background noise into consideration. Here we present several major updates for XBSeq. Firstly, we update the background annotation files (utilizing the same procedures as given in [8]) needed for measuring background noise for human and mouse organism of various genome builds. Secondly, we incorporate functionalities of Rsubread and GenomicRanges packages to enable direct processing of alignment files (.bam) within R environment. Thirdly, besides the non-parametric method for estimation of parameters proposed by the original paper, we provide one additional method for estimating parameters by using maximum likelihood estimation (Eq. 2). Fourthly, we provide a beta distribution approximation method for testing DEGs, which is much faster in speed and more memory efficient compared to the original statistical method (Eq. 11). Fifthly, XBSeq2 now supports APA differential usage inference by using the functionalities provided by roar package. The background annotation file as well as the APA annotation file for various genome builds are available to download from github: https://github.com/Liuy12/XBSeq_files.

### Discrimination between DE and non-DE genes

In order to compare XBSeq2 with edgeR, DESeq2 and XBSeq, we generated synthetic datasets where we can control the differential expression status of each gene by following the procedure described in the methods section. Basically, 5000 genes and their corresponding background noise were firstly simulated from negative binomial and Poisson distribution respectively with parameters estimated from a real RNA-seq dataset after discarding genes with relatively low mapped reads or larger dispersion (top 10%). We showed that by discarding genes with high dispersions, we did not introduce bias towards to a certain method (Additional file 1: Table S4). 500 genes were randomly selected to be differentially expressed with 1.5-fold change. Background noise with different dispersion levels was simulated. All statistical metrics were calculated based on the average of 100 simulations.

**Fig. 1:**
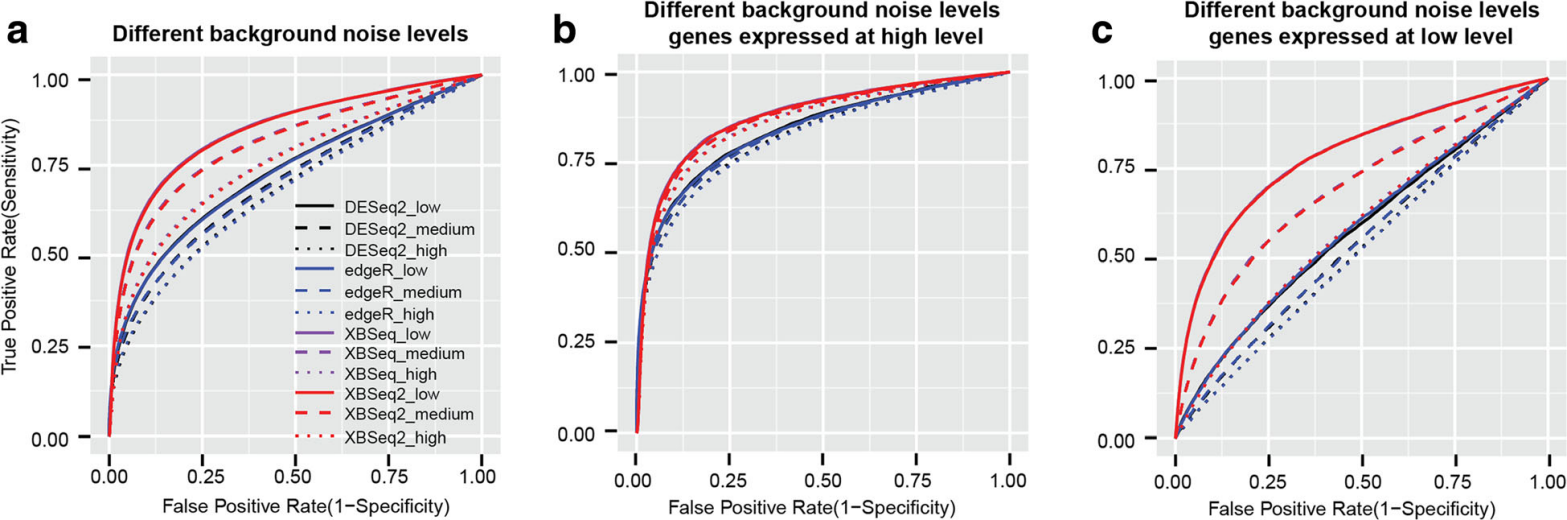
ROC curves of different methods under various levels of background noise. ROC curves of DESeq2, edgeR, XBSeq, XBSeq2 under low, intermediate or high level of background noise (**a**); ROC curves of different methods but only with highly expressed genes (genes above 75% quantile of expression intensity) (**b**); ROC curves of different methods but only with genes expressed at low levels (genes below 25% quantile of expression intensity) (**c**); Simulations were carried out 100 times and average AUC were used. Dataset with 3 number of replicates per condition, 10% DEGs with 1.5-fold change was used

We compared different methods for their ability to discriminate between differentially expressed genes and non-differentially expressed genes by examining area under the ROC curve. As shown in Fig. 1 & Additional file 1: Table S1, in general, XBSeq2 and XBSeq perform better than the other two methods with larger AUCs. To be specific, when background noise is at a low level, XBSeq2 achieved an AUC of 0.84 which is very close to XBSeq (AUC: 0.85), while AUCs for DESeq2 and edgeR are both 0.73. When we increased the dispersion level of background noise, all four methods have decreased AUCs. XBSeq2 and XBSeq are still the best methods with AUCs 0.75 under high background noise compared to DESeq2 and edgeR (AUC: 0.68 for DESeq2, 0.67 for edgeR). We also investigated the performance of different methods separately for genes with either high (> 75% quantile) or low (< 25% quantile) expression level. As shown in Fig. 1b and c, for genes with relatively high expression intensity, XBSeq and XBSeq2 still perform equally well (AUC = 0.88 for both under low background noise) and only slightly better than DESeq2 and edgeR (AUCs, 0.84 for both under low background noise). On the other hand, for genes with relatively low expression intensity, XBSeq and XBSeq2 perform much better than DESeq2 and edgeR under low background noise (AUCs, 0.78 for XBSeq and XBSeq2, 0.58 for DESeq2 and edgeR). However, all methods show poor performance for genes with relatively low expression under high background noise (AUCs, 0,58 for XBSeq and XBSeq2, 0.52 for DESeq2 and edgeR). Also, we evaluated the MLE-based method for parameter estimation compared to the original non-parametric based method. As shown in Additional file 1: Table S2, both non-parametric (NP) based estimation and maximum likelihood estimation (MLE) based estimation showed better performance than DESeq2 with larger area under the ROC curve (AUC). NP-based estimation has slightly better performance than MLE-based estimation when samples number is smaller than 10. When sample number is big enough, there seems to be no difference in terms of performance. Last but not least, we evaluated the parameter *big_count*, which defines the cutoff for genes with large counts. As shown in Additional file 1: Table S3, the parameter only has a slight influence on the performance of XBSeq2, which indicates that beta distribution approximation test has similar performance compared to the original statistical test. Overall, XBSeq2 performs equally with XBSeq in terms of AUC under various conditions and both methods perform better than DESeq2 and edgeR, especially for genes with relatively low expression intensity.

### Control of false discoveries

**Fig. 2:**
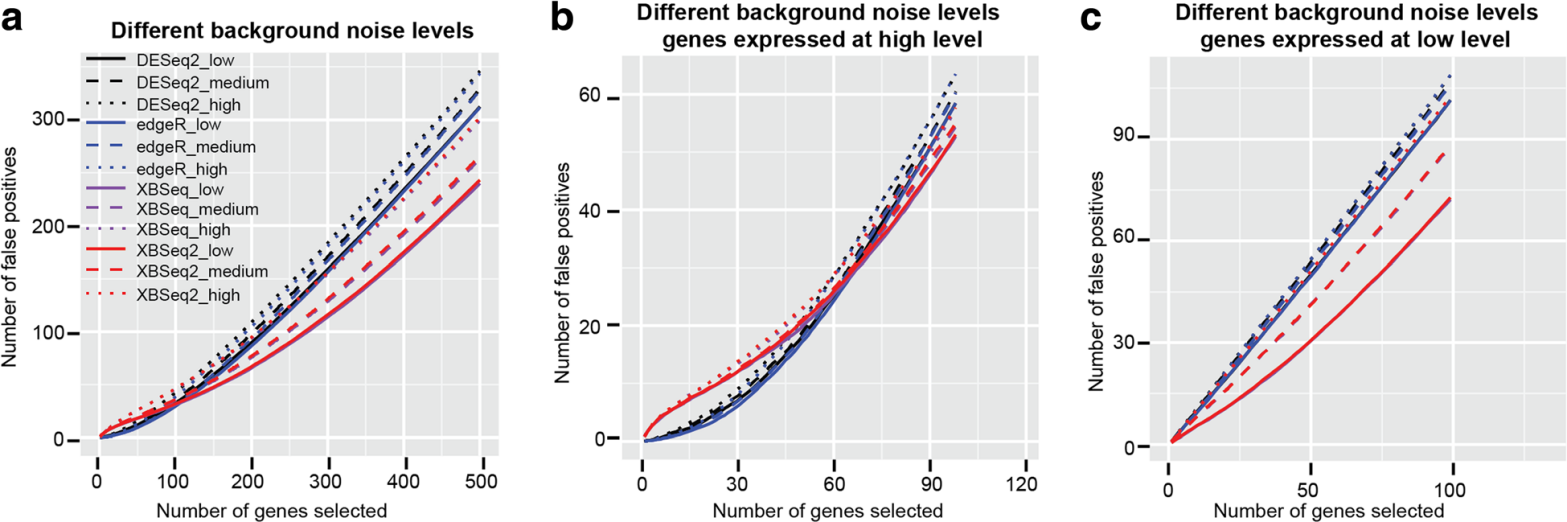
False discovery curves different methods under various levels of background noise. False discovery curves of DESeq2, edgeR, XBSeq, XBSeq2 under low, intermediate or high level of background noise (**a**); False discovery curves of different methods but only with highly expressed genes (genes above 75% quantile of expression intensity) (**b**); False discovery curves of different methods but only with genes expressed at low levels (genes below 25% quantile of expression intensity) (**c**); Simulations were carried out 100 times and average number of false discoveries were used. Dataset with 3 number of replicates per condition, 10% DEGs with 1.5-fold change was used

We also compared the different methods in terms of the number of false discoveries encountered among top ranked differentially expressed genes based on *p* value. As shown in Fig. 2 & Additional file 1: Table S1, overall, XBSeq2 and XBSeq perform better than DESeq2 and edgeR. To be specific, under low background noise, XBSeq2 identified 243 number of false discoveries out of 500, which is comparably well to XBSeq (# of FDs, 240). Both methods perform better than DESeq2 and edgeR (# of FDs, 313 and 312 respectively). With increased background noise, all four methods detect an increased number of false discoveries. We then compared the performance of different methods separately for genes expressed at high and low levels as we did earlier. For genes with relatively high expression, XBSeq and XBSeq2 only perform slightly better than DESeq2 and edgeR (# of FDs, 53 for XBSeq and XBSeq2, 58 for DESeq2 and edgeR). However, for genes expressed at low levels, XBSeq and XBSeq2 performed much better than DESeq2 and edgeR under low background noise (# of FDs, 72 for XBSeq, 73 for XBSeq2, 102 for DESeq2, 101 for edgeR). Overall, XBSeq2 performs equally with XBSeq in terms of number of false discoveries under various conditions and both methods perform better than DESeq2 and edgeR, especially for genes expressed at low levels.

### Statistical power

**Fig. 3:**
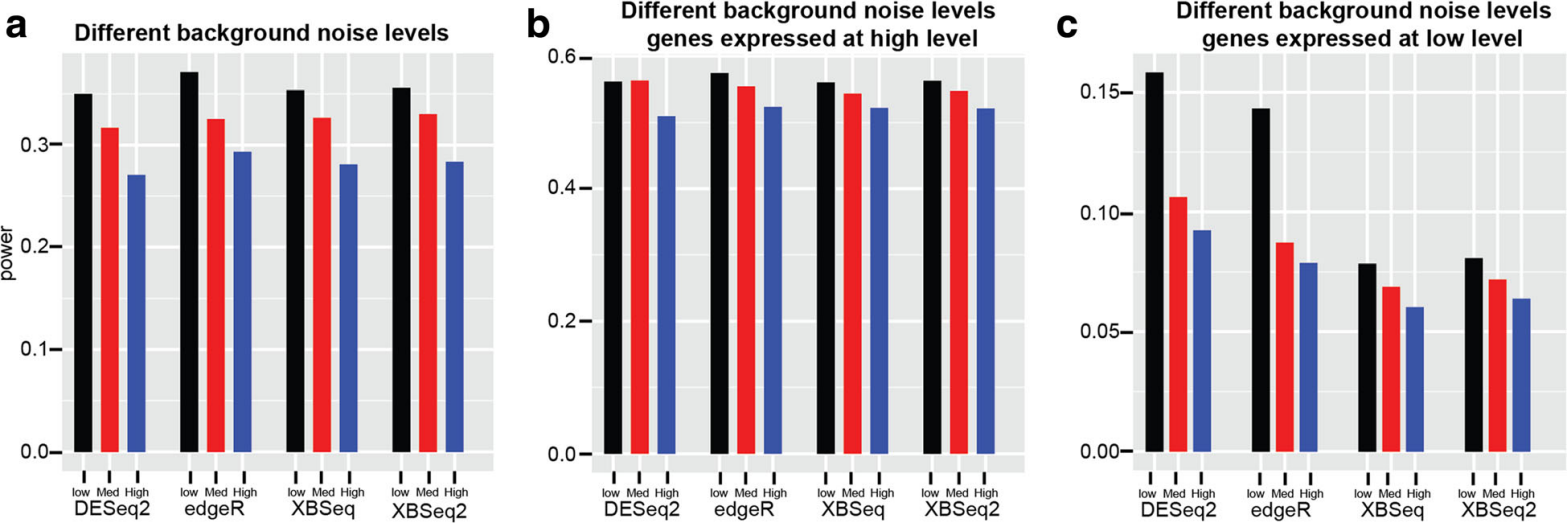
Statistical power of different methods under various levels of background noise. Bar chart of statistical power for DESeq2, edgeR, XBSeq, XBSeq2 under low, intermediate or high level of background noise (**a**); Bar chart of statistical power for different methods but only with highly expressed genes (genes above 75% quantile of expression intensity) (**b**); Bar chart of statistical power for different methods but only with genes expressed at low levels (genes below 25% quantile of expression intensity) (**c**); Simulations were carried out 100 times and average statistical power were used. Dataset with 3 number of replicates per condition, 10% DEGs with 1.5-fold change was used

We compared the different methods in terms of statistical power achieved at a pre-selected *p* value cutoff (*p* value = 0.05). As shown in Fig. 3 & Additional file 1: Table S1, overall, all four methods have similar statistical power with edgeR slightly better than other methods (Power, 0.35 for XBSeq, 0.36 for XBSeq, 0.35 for DESeq2, 0.37 for edgeR under low background noise). And all methods have decreased statistical power when the dispersion of background noise is increased. We also compared different methods separately for genes expressed at high and low levels as we did earlier. As shown in Fig. 3b and c, all four methods achieved similar statistical power for highly expressed genes. For genes expressed at low levels, DESeq2 and edgeR perform better than XBSeq and XBSeq2 (Power, 0.16 for DESeq2, 0.14 for edgeR, 0.08 for XBSeq and XBSeq2 under low background noise). However, when background noise is increased, all methods exhibit poor performance with similar statistical power for genes expressed at low levels. Overall, XBSeq2 perform comparably well with other methods regarding statistical power.

### Identify APA events from RNA-seq dataset derived from ccRCC tumors and adjacent normal tissues

By utilizing XBSeq2 algorithm, we carried out differential APA usage analysis and differential expression analysis with RNA-seq samples derived from ccRCC tumors and adjacent normal tissues (see Methods Section). APA annotation was generated by using POLYA program as described in methods section. In total, we identified 179 number of genes with differential APA usage with roar value (ratio of ratios), fold change, larger than 1.5, average expression intensity above second quantile of total genes and adjusted *p* value smaller than 0.1. MYH9, one of the top-ranked genes with differential APA usage, has been previously demonstrated to be associated with end-stage renal disease in African Americans [23]. Then we proceeded to identify DEGs between the two groups using XBSeq2. In total, we identified 417 number of genes that are differentially expressed between tumor and adjacent normal samples with a fold change larger than 1.5, average expression intensity above second quantile of total genes and adjusted *p* value smaller than 0.1. We also compared the DEGs identified by XBSeq2, DESeq2 and edgeR (Additional file 1: Figure S1). 399 out of 417 DEGs identified by XBSeq2 are also identified by DESeq2 and edgeR. Intriguingly, only two of the genes we identified earlier with differential APA usage were found to be differentially expressed, PAG1 and FAM171A1, which might indicate that regulation through APA usage is independent of regulation through gene expression level.

## Discussion

**Fig. 4:**
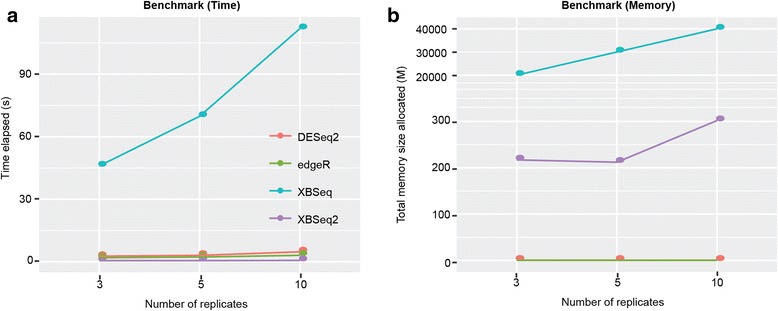
Benchmark of different methods under low level of background noise. Benchmark of DESeq2, edgeR, XBSeq, XBSeq2 in terms of computation time (**a**); and total number of computational memory allocated (**b**). Methods were benchmarked with datasets of 3, 5, or 10 number of replicates per condition, 10% DEGs with 1.5-fold change. Benchmark procedure was carried out under MacBook Pro, 2.7 GHz Intel Core i5, 8 GB 1867 MHz DDR3

In this paper, we present several major updates to XBSeq, a method we previously developed for testing differential expression for RNA-seq. In order to compare different statistical methods for their ability to correctly identify DEGs, we carried out simulation studies to generate synthetic RNA-seq datasets with different levels of background noise. While Flux Simulator algorithm [24] provides a simulation path starting from the very beginning, in this report, we directly simulate the expression level with Negative Binomial and Poisson distribution for signal and noise expression levels, allowing us to efficiently estimate the accuracy of our proposed algorithm. Sequencing Quality Control (SEQC) project provide unique resources for comprehensive evaluating RNA-seq accuracy, reproducibility and information content [25]. However, the background noise for SEQC data cannot be simply quantified, which makes it difficult for evaluating algorithms under different background noise. Taking all these into consideration, we decided to apply similar simulation procedure as XBSeq [8]. As shown in the results section, XBSeq2 performed equally well with XBSeq and both performed better than DESeq2 and edgeR in terms of AUC (Fig. 1) and number of FD (Fig. 2). For statistical power (Fig. 3), all four methods have similar performance with edgeR being slightly better. Finally, we benchmarked all the methods with regard to time and memory consumption. As shown in Fig. 4, XBSeq2 consumes the least amount of time compared to other three methods and also has a significant increase in efficiency compared to XBSeq. Taken together, XBSeq2 and XBSeq are robust against background noise and provide more accurate detection of DEGs. In addition, XBSeq2 are faster and more memory efficient than XBSeq.

We incorporated functionalities for testing differential APA usage from roar package. As we mentioned earlier, DaPars is one novel algorithm for de novo identification and quantification of dynamic APA events between tumor and matched normal tissues, regardless of any prior APA annotation. To the contrary, roar do need user to provide APA annotation and lacks the ability to identify novel APA sites. The only reason we incorporate roar instead of DaPars, is for programming language compatibility. We demonstrated the functionality of XBSeq2 for testing differential APA usage by using our in-house CCRCC dataset. We found 179 genes with differential APA usage. Interestingly, only 2 out of the 179 genes were found to be differentially expressed between tumor and normal samples. It could be that the APA annotation we generated is far from complete and some novel APA sites might be overlooked. Another possible explanation is that APA usage regulate transcriptomic activity through a different mechanism without affecting gene expression intensity.

## Conclusions

We presented the latest updates of XBSeq in this report. The updated XBSeq2 package provide a much fast execution time and implemented in a computer memory efficient manner to allow user to process data directly from BAM files, much fast for testing differential expression for RNA-seq datasets, as well as a new functions, within one XBSeq2 package to identify differential APA usage. XBSeq2 is available from Bioconductor: http://bioconductor.org/packages/XBSeq/.

## Additional file

Additional file 1: Figures and Tables to provide additional analysis results. (PDF 310 kb)

## Abbreviations

APA: Alternative polyadenylation
AUC: Area under ROC curve
BFGS: Broyden–Fletcher–Goldfarb–Shanno
CCRCC: clear cell renal cell carcinoma
DE: differential expression
DEGs: differentially expressed genes
EM: expectation maximization
MLE: maximum likelihood estimation
NGS: next generation sequencing
RNA-seq: RNA sequencing
ROC: receiver operating characteristic
UTR: untranslated regions
