# Supplementary material for: XBSeq2: a fast and accurate quantification of differential expression and differential polyadenylation
Source: BMC Bioinformatics. 2017 Oct 3;18(Suppl 11):384. doi: 10.1186/s12859-017-1803-9 (PMC5629564; doi:10.1186/s12859-017-1803-9)
Supplement: Additional file 1: — Figures and Tables to provide additional analysis results. (PDF 310 kb) [file 12859_2017_1803_MOESM1_ESM.pdf]

XBSeq2: a fast and accurate quantification of differential expression and differential Polyadenylation

Yuanhang Liu, Ping Wu, Jingqi Zhou, Teresa L Johnson-Pais, Zhao Lai, Ronald Rodriguez, Yidong Chen

Supplementary Figures

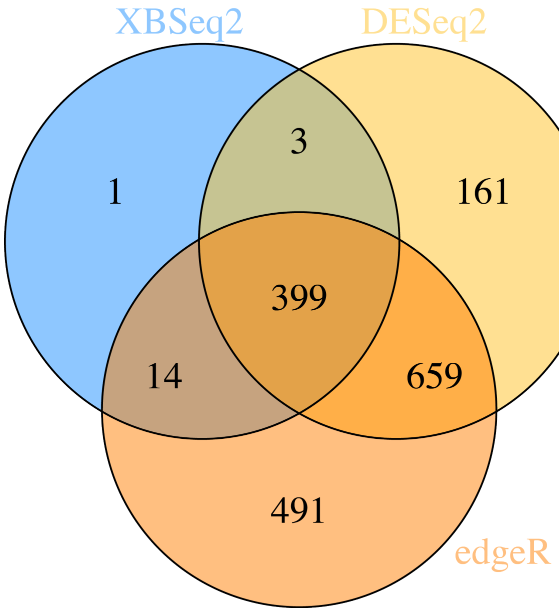

Figure S1. Venn diagram of DEGs identified by XBSeq2, edgeR and DESeq2 for CCRCC dataset.

Supplementary Tables

		DESeq2				edgeR				XBSeq				XBSeq2		
# of samples/group		Low	Interm	High		Low	Interm	High		Low	Inter m	High		Low	Inter m	High
3 replicates	AUC	0.727	0.702	0.678		0.726	0.70	0.675		0.846	0.81	0.753		0.844	0.807	0.75

all genes	# of FD	312.5	329.3	346		311.9	327.6	343.5		240	263.2	300		243.1	266	302.4
	Power	0.35	0.317	0.271		0.371	0.326	0.293		0.354	0.327	0.281		0.356	0.33	0.284
3 replicates	AUC	0.839	0.834	0.819		0.838	0.832	0.817		0.878	0.872	0.86		0.876	0.87	0.858
Highly expressed genes	# of FD	58.45	60.47	63.36		58.48	60.23	63.44		52.69	54.35	57.12		53.02	54.97	57.69
	Power	0.561	0.563	0.51		0.574	0.554	0.524		0.56	0.544	0.522		0.563	0.548	0.521
3 replicates	AUC	0.576	0.541	0.519		0.581	0.542	0.517		0.785	0.691	0.585		0.784	0.688	0.583
Lowly expressed genes	# of FD	101.6	106.7	108.8		101.5	106.1	108.9		72.15	87.82	102.4		72.78	88.29	102.6
	Power	0.158	0.106	0.092		0.143	0.087	0.079		0.078	0.069	0.06		0.081	0.072	0.064

Table S1. AUC, # of false discoveries and statistical power of DESeq2, edgeR, XBSeq and XBSeq2 under low, intermediate or high level of non-exonic mapped reads as shown in Figure 1,2&3.

nreps	2	5	10	20	50	100
DESeq2	0.6133764	0.6918067	0.7660507	0.8077591	0.8568991	0.9249884
XBSeq2_NP	0.6781787	0.7904809	0.8393582	0.9038551	0.9453244	0.9678244
XBSeq2_MLE	0.6639529	0.7855071	0.839628	0.9038982	0.9453244	0.9678244

Table S2. AUC of DESeq2, XBSeq2_NP (non-parametric estimation), XBSeq2_MLE (maximum likelihood estimation) with different number of replicates (2, 5, 10, 20, 50, 100), 10 percent of DE genes, 1.5 fold change.

big_count	0	100	500	1000	5000	10000
XBSeq2	0.7326849	0.7326849	0.7326849	0.7326849	0.7326849	0.7326849
XBSeq2_big	0.7303831	0.7301907	0.731404	0.7297004	0.7298667	0.7318849

Table S3. AUC of XBSeq2 with different cutoff for big_count parameter (a beta distribution approximation will be applied to identify DEGs for genes with expression larger than big_count), 10 percent of DE genes, 1.5 fold change, 3 number of replicates.

Filter on dispersion	DESeq2	edgeR	XBSeq	XBSeq2
Yes	0.686	0.678	0.763	0.762
No	0.687	0.681	0.745	0.743

Table S4. AUC of DESeq2, edgeR, XBSeq, XBSeq2 when we do or don't discard top 15% highly dispersed genes during simulation procedure, 10 percent of DE genes, 1.5 fold change, 3 number of replicates
